# Cutaneous infection by non-diphtheria-toxin producing and penicillin-resistant *Corynebacterium diphtheriae* strain in a patient with diabetes mellitus

**DOI:** 10.1099/acmi.0.000284

**Published:** 2021-11-30

**Authors:** Max Roberto Batista Araújo, Mireille Ângela Bernardes Sousa, Luisa Ferreira Seabra, Letícia Aparecida Caldeira, Carmem Dolores Faria, Sérgio Bokermann, Lincoln Oliveira Sant’Anna, Louisy Sanches dos Santos, Ana Luíza Mattos-Guaraldi

**Affiliations:** ^1^​ Operational Technical Nucleus, Microbiology, Hermes Pardini Institute. Av. das Nações, 3801 - Parque Jardim Itaú, Minas Gerais, Brazil; ^2^​ Bacterial and Fungal Diseases Service, Ezequiel Dias Foundation, Belo Horizonte, Minas Gerais, Brazil; ^3^​ Center of Bacteriology, Adolfo Lutz Institute, Secretary of Health of the State of São Paulo, Brazil; ^4^​ Laboratory of Diphtheria and Corynebacteria of Clinical Relevance, Faculty of Medical Sciences, Rio de Janeiro State University, The Collaborating Center for Reference and Research on Diphtheria, National Health Foundation, Ministry of Health, Rio de Janeiro, Brazil

**Keywords:** *Corynebacterium diphtheriae*, cutaneous diphtheria, diabetes, diphtheria toxin, MALDI-TOF MS, penicillin-resistance

## Abstract

Diphtheria is a potentially fatal infection, mostly caused by diphtheria toxin (DT)-producing *Corynebacterium diphtheriae* strains. During the last decades, the isolation of DT-producing *C. diphtheriae* strains has been decreasing worldwide. However, non-DT-producing *C. diphtheriae* strains emerged as causative agents of cutaneous and invasive infections. Although endemic in countries with warm climates, cutaneous diphtheria is rarely reported in Brazil. Presently, an unusual case of skin lesion in a Brazilian elderly diabetic patient infected by a penicillin-resistant non-DT-producing *C. diphtheriae* strain was reported. Laboratory diagnosis included mass spectrometry and multiplex PCR analyses. Since cutaneous diphtheria lesions are possible sources of secondary diphtheria cases and systemic diseases and considering that penicillin is the first line of antimicrobial agent for the treatment of these infections, the detection of penicillin-resistant strains of diphtheria bacilli should be a matter of concern. Thus, cases similar to the presently reported should be appropriately investigated and treated, particularly in patients with risk factor (s) for the development of *C. diphtheriae* invasive infections, such as diabetes. Moreover, health professionals must be aware of the presence of *C. diphtheriae* in cutaneous lesions of lower limbs, a common type of morbidity in diabetic patients, especially in tropical and subtropical countries.

## Introduction

Diphtheria is a highly contagious infectious disease that often affects the respiratory tract and the skin, mostly caused by diphtheria toxin (DT)-producing *Corynebacterium diphtheriae* strains. Although included among vaccine-preventable diseases, diphtheria remains occurring worldwide, including in Brazil [1–5], leading to death even in immunized individuals [5–7].

Cutaneous diphtheria is normally associated with colonization of pre-existing skin lesions, such as surgical wounds, burns, and insect bites, mostly on the legs, feet, and hands, by both DT-producing and non-DT-producing *C. diphtheriae* strains [3, 8–10]. *C. diphtheriae *-infected lesions act as reservoirs of this pathogen that can contaminate the environment and induce human infections in contacts more efficiently than pharyngeal infections [8, 11], contributing to the emergence of outbreaks and epidemics in vulnerable populations [11, 12].

In addition, to associated with skin infections, non-DT-producing diphtheria strains have been also reported as agents of invasive diseases, such as endocarditis, pneumonia, osteomyelitis and catheter-related infections, mainly in adult patients, with several cases of death [3, 5, 13–15], indicating the expression of virulence mechanisms other than DT production [3, 13, 14, 16–18].

Penicillin and erythromycin have long been the drugs of choice for the treatment of diphtheria and other *C. diphtheriae* infections. However, drug resistance is a matter of concern worldwide, especially due to the increase of reported cases in the last years [2, 19–22]. Considering the importance of continuous surveillance of diphtheria cases and the emergence of drug-resistant *C. diphtheriae* clones, the present work aims to report the clinical and microbiological aspects of a case of cutaneous infection caused by penicillin-resistant non-DT-producing *C. diphtheriae* strain in a Brazilian diabetic patient.

## Case report

In December 2018, a female patient, non-immunized against diphtheria, with a history of type I diabetes mellitus was seen by her general practitioner in Minas Gerais State, Brazil. The patient was diagnosed with a left leg injury characterized by a thin membrane, fissures, and secretion. In January 2019, the patient went under medical supervision and began treatment with ciprofloxacin for 8 days, combined with cyanocobalamin, pyridoxine hydrochloride, thiamine nitrate, and sodium diclofenac; oral use of diosmin and hesperidin; topical use of silver sulfadiazine 1 % and cerium nitrate 2.2 %. Although treatment led to the significant healing of cutaneous lesions, 2 months later, skin infections reappeared with a membrane formation. Swabs from lesions were, then, collected and sent to laboratory analysis.

**Fig. 1.**
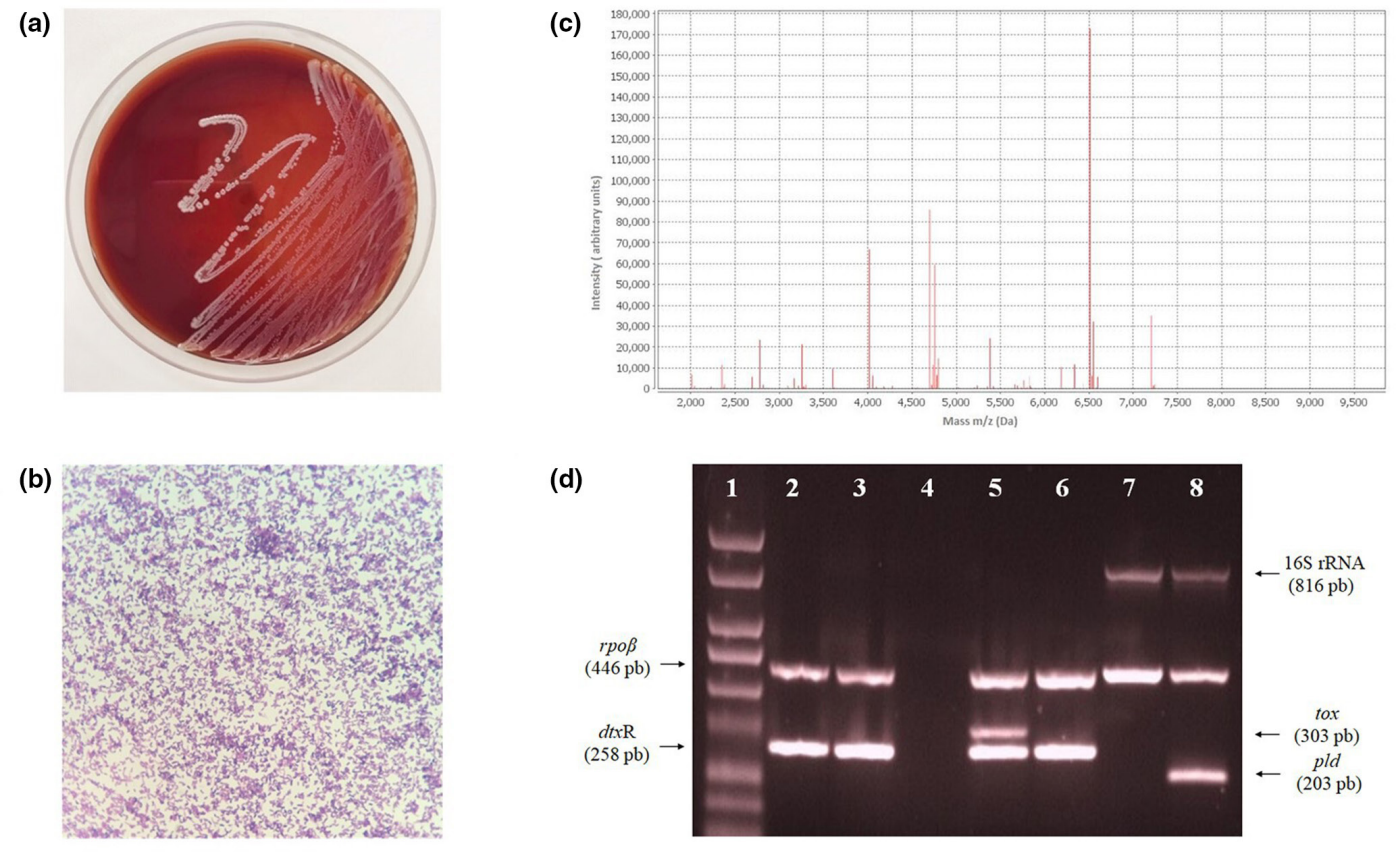
Microbiological features of penicillin-resistant *Corynebacterium diphtheriae* strain isolated of infected skin ulcer from a Brazilian diabetic elderly female patient. (**a**) Colonial morphology on 5 % sheep blood agar plate. (**b**) Gram-staining (original magnification,×1000) showing pleomorphic Gram-positive bacillary forms. (**c**) MALDI-TOF MS spectrum. (**d**) Amplification profile by multiplex PCR assay for differentiation between *C. diphtheriae* (including *Corynebacterium belfantii* and *Coryrnebacterium rouxii*), *Corynebacterium ulcerans* and *Corynebacterium pseudotuberculosis* strains and detection of diphtheria toxin gene (*tox*): Lane 1, molecular weight (1 kb DNA ladder); Lanes 2 and 3, *C. diphtheriae* clinical isolate (*tox^-^*); Lane 4, negative control (reaction without template DNA); Lane 5, *C. diphtheriae* ATCC 27012 (*tox^+^*); Lane 6, *C. diphtheriae* ATCC 27010 (*tox^-^*); Lane 7, *C. ulcerans* 809 (*tox^-^*); Lane 8, *C. pseudotuberculosis* ATCC 19410 (*tox^-^*).

Culture of swab were performed on 5 % sheep’s blood agar (bioMérieux^®^, Brazil). After incubation at 37 °C for 48 h, the growth of white, opaque colonies, showing slight hemolysis, was observed (Fig. 1a). Gram-stained optical microscopy of these colonies showed Gram-positive bacillary forms, arranged in pallid shapes with angular formations between cells (Fig. 1b). The Matrix-Assisted Laser Desorption/Ionization Time-of-Flight Mass Spectrometry (MALDI-TOF MS) analysis in the semi-automated system VITEK^®^ MS (bioMérieux^®^) identified this bacterial isolate as *C. diphtheriae* with 99 % probability. The obtained mass spectra are processed by a specific software, namely MYLA^®^ (bioMérieux^®^, France), and compared to the database containing the reference spectra or ‘super spectra’. The VITEK MS (bioMérieux^®^) instrument compares these spectra with the Spectral Archive and Microbial Identification System (SARAMIS^®^, bioMérieux^®^, France) database, which in turn uses common peaks of strains of the same species (between 15–20) to build a ‘super spectrum’. Finally, agreement values above 60 % mean the species was identified. The MALDI-TOF MS spectrum of *C. diphtheriae* clinical isolate can be observed in Fig. 1(c). In addition, two other colonies grown in the primary culture were identified by MS MALDI-TOF as *Staphylococcus aureus* and *Pseudomonas putida *.

The clinical isolate was sent to the Brazilian Ministry of Health Laboratories, Adolfo Lutz Institute and Laboratory of Diphtheria and Corynebacteria of Clinical Relevance (LDCIC), in order to confirm *C. diphtheriae* identification, investigate the DT-production by multiplex Polymerase Chain Reaction (mPCR) assays and determine the susceptibility to antimicrobials. mPCR assays were carried out as previously described with primers pairs targeting the following genes: *rpo*B - β subunit of RNA polymerase - of *C. diphtheriae* (including the novel species *Corynebacterium belfantii* and *Corynebacterium rouxii *), *Corynebacterium ulcerans* and *Corynebacterium pseudotuberculosis* (C2700F and C3130R, 446 bp); 16S rRNA - 16S ribosomal RNA - of both *C. pseudotuberculosis* and *C. ulcerans* (16SF and 16 SR; 816 bp); *dtx*R - diphtheria toxin repressor - of *C. diphtheriae* (DtxR1F and DtxR1R, 258 bp); *pld* - sphingomyelinase - of *C. pseudotuberculosis* (pldF and pldR2, 203 bp); *tox* - diphtheria toxin (Dipht 4F and Dipht 4R, 303 bp) [23, 24]. As illustrated in Fig. 1(d), mPCR results confirmed that the clinical isolate was a non-DT producing *C. diphtheriae* strain (positive for *rpo*B and *dtx*R genes; negative for *tox*, 16S rRNA and *pld* genes).

Additionally, the antimicrobial profile of the clinical isolate was determined using the disc-diffusion method [25] according to the guideline provided by the Brazilian Committee for Antimicrobial Susceptibility Testing (BrCAST) [26]. Bacterial suspension of the isolate was prepared in saline with the turbidity equivalent to the 0.5 McFarland scale and seeded on Mueller Hinton Agar plate supplemented with 5 % defibrinated horse’s blood and 20 mg l^−1^ β-NAD (PlastLabor^®️^, Brazil). Then, the following antibiotics (DME^®^, Brazil) were transferred to the surface of the seeded plate: benzylpenicillin (1U), ciprofloxacin (5 µg), gentamicin (10 µg), moxifloxacin (5 µg), rifampicin (5 µg), tetracycline (30 µg), and vancomycin (5 µg). Results were obtained after incubation in 5 % CO_2_ atmosphere at 35±1 °C for 40–44 h. The interpretation of susceptibility was performed according to the breakpoints established by the BrCAST guideline. The quality control of the tests was also carried out as recommended by the BrCAST document and included the use of *Streptococcus pneumoniae* ATCC 49619 strain. *C. diphtheriae* clinical isolate showed resistance to benzylpenicillin and gentamicin.

Despite the communication of the result to the medical, the established treatment was not based on antibiotics and included: betamethasone dipropionate and betamethasone disodium phosphate by using three doses per 8 days; and dry pinus pinaster Aiton extract and fludroxicortide 0.125 mg g^−1^ twice daily. Fortunately, there was observed a remission of membrane and skin infection.

## Discussion

Diabetes mellitus is a major chronic disease that continues to increase significantly. One of the most important and costly complications of diabetes is cutaneous ulceration that may be colonized by pathogenic and drug resistant bacteria. Bacterial colonization and/or infection often impair treatment success and may be responsible for ulcers chronicity [27]. Therefore, continued clinical and microbiological vigilance of lesions remain necessary.

Some *Corynebacterium* spp. and others irregular Gram-positive rods (IGPR) are common colonizers of skin and mucosal surfaces. Due to this, and the fact that identification of IGPR through conventional biochemical tests used to be difficult for most microbiology laboratories, IGPR isolated from non-respiratory clinical sites used to be discarded as contaminants until recently. However, with the emergence of MALDI-TOF-MS as a method for microbial identification in many clinical laboratories, the majority of these IGPR can now be routinely identified to the species level. Consequently, IGPR has been found clinically significant in some types of infections [28, 29]. At the same time, non-DT-producing *C. diphtheriae* strains emerged as causative agents of invasive infections in several countries and now it is more widely accepted that they can also cause skin and wound infections [29]. These infections are sometimes difficult to clinically distinguish from other skin infections [3, 30]. Thus, swabbing of the lesion is essential [11].

Although DT-mediated systemic manifestations are not expected as a result of cutaneous infections caused by a non-DT-producing *C. diphtheriae* isolate, development of respiratory diphtheria and invasive infections were reported in various opportunities, independent of the toxigenic status of *C. diphtheriae* strain [10, 11, 30–32]. Thus, the patient, as well as close contacts, should be monitored. People who are at increased risk for *C. diphtheriae* dissemination include those with comorbidities, such as HIV and diabetes, a history of alcohol abuse or intravenous drug use and those that live in crowded or unsanitary conditions [10, 33].

Besides the implementation of isolation precautions, medical management for *C. diphtheriae* cutaneous infections often includes the use of antibiotics, mostly erythromycin or penicillin. However, penicillin-resistant *C. diphtheriae* strains have been increasingly detected from human infections, including cutaneous diphtheria [19, 21, 22, 34]. Therefore, antimicrobial susceptibility testing for diphtheria bacilli should be mandatory.

Presently, a case of cutaneous lesion infected by two known human pathogens, *C. diphtheriae* and *S. aureus *, were reported. Co-infections with *S. aureus *, also *Streptococcus pyogenes *, are commonly described in human cases of cutaneous diphtheria. Although the patient had not to be immunized against diphtheria and had one of the risk factors for systemic diseases due to non-DT-producing *C. diphtheriae* strains, the patient monitoring was not performed, and the treatment did not include any antimicrobial. Cases of infections due to penicillin-resistant *C. diphtheriae* strains, independent of DT-production, should be a matter of concern since this is the recommended first-line agent for the treatment and prophylaxis of diphtheria and other *C. diphtheriae* infections [22].

Considering that *C. diphtheriae* cutaneous infections can present a possible source of secondary diphtheria cases and systemic diseases, they need to be properly investigated, treated, and reported. Data emphasize that microbiologists should not promptly discard colonies of IGPR from cultures, even when grown associated with one or more potentially pathogenic species, especially from patients of risk groups for *C. diphtheriae* infections in tropical and/or developing countries. Moreover, this study highlighted that health professionals must keep aware of the presence of *C. diphtheriae *, especially in cutaneous lesions of lower limbs, a common type of morbidity in diabetic patients, independently of the following aspects: DT-production, age, complete immunization status and health conditions. Finally, this emphasizes the importance of carry out the antimicrobial susceptibility test before the institution of antimicrobial therapy.
